# Typical features of Parkinson disease and diagnostic challenges with microdeletion 22q11.2

**DOI:** 10.1212/WNL.0000000000005660

**Published:** 2018-06-05

**Authors:** Erik Boot, Nancy J. Butcher, Sean Udow, Connie Marras, Kin Y. Mok, Satoshi Kaneko, Matthew J. Barrett, Paolo Prontera, Brian D. Berman, Mario Masellis, Boris Dufournet, Karine Nguyen, Perrine Charles, Eugénie Mutez, Teodor Danaila, Aurélia Jacquette, Olivier Colin, Sophie Drapier, Michel Borg, Ania M. Fiksinski, Elfi Vergaelen, Ann Swillen, Annick Vogels, Annika Plate, Claudia Perandones, Thomas Gasser, Kristien Clerinx, Frédéric Bourdain, Kelly Mills, Nigel M. Williams, Nicholas W. Wood, Jan Booij, Anthony E. Lang, Anne S. Bassett, On behalf of the International Research Group on 22q11.2DS-associated Parkinson's Disease

**Affiliations:** From The Dalglish Family 22q Clinic for Adults and Department of Psychiatry (E.B., A.M.F., A.S.B.), Toronto General Research Institute (A.S.B.), and Division of Cardiology, Department of Medicine (A.S.B.), University Health Network, Toronto, Canada; De Hartekamp Groep (E.B.), Centre for People with Intellectual Disability, Haarlem; Department of Nuclear Medicine (E.B., J.B.), Academic Medical Center, Amsterdam, the Netherlands; Clinical Genetics Research Program and Campbell Family Mental Health Research Institute (N.J.B., A.M.F., A.S.B.), Centre for Addiction and Mental Health, Toronto; Institute of Medical Science (N.J.B., M.M., A.E.L., A.S.B.), Division of Neurology, Department of Medicine (C.M., M.M., A.E.L.), and Department of Psychiatry (A.S.B.), University of Toronto; Deer Lodge Movement Disorders Centre (S.U.); Section of Neurology (S.U.), Division of Internal Medicine, Rady Faculty of Health Sciences, University of Manitoba, Winnipeg; Morton and Gloria Shulman Movement Disorders Centre and the Edmond J. Safra Program in Parkinson's Disease Research (C.M., A.E.L.), Toronto Western Hospital and University of Toronto, Canada; Department of Molecular Neuroscience (K.Y.M., N.W.W.), UCL Institute of Neurology, London, UK; Department of Neurology (S.K.), Kansai Medical University, Osaka, Japan; Department of Neurology (M.J.B.), University of Virginia School of Medicine, Charlottesville; Medical Genetics Unit (P.P.), Perugia University Hospital, Italy; Department of Neurology (B.D.B.), University of Colorado Anschutz Medical Campus, Aurora; Neurology Section (B.D.B.), VA Eastern Colorado Health Care System, Denver; Cognitive & Movement Disorders Clinic and Hurvitz Brain Sciences Research Program (M.M.), Sunnybrook Health Sciences Centre, Toronto, Canada; Departments of Clinical Neurosciences (Movement Disorders) (B.D.) and Genetics (Neurogenetics) (K.N.), Timone University Hospital (AP-HM), Provence-Alpes-Côte d’Azur; Aix-Marseille University (B.D., K.N.), Marseille; Department of Genetics (Neurogenetics) (P.C., A.J.), Pitié-Salpêtrière University Hospital; Sorbonne University (P.C., A.J.), Paris; Department of Neurosciences (Movement Disorders) (E.M.), Lille University Hospital; Lille University (E.M.); Department of Neurology (Movement Disorders) (T.D.), Pierre Wertheimer University Hospital, Lyon; Marc Jeannerod Center for Cognitive Neurosciences (T.D.), Lyon-1 University; Department of Neurology (Movement Disorders) and Clinical Investigation Center (Clinical and Experimental Neurosciences) (O.C.), Poitiers University Hospital; Department of Neurology (Movement Disorders) (S.D.), Rennes University Hospital; Rennes-1 University (S.D.); Department of Clinical Neurosciences (Movement Disorders) (M.B.), Nice University Hospital, France; Department of Psychiatry (A.M.F.), Rudolf Magnus Institute of Neuroscience, University Medical Center Utrecht, the Netherlands; Center for Human Genetics (E.V., A.S., A.V.), University Hospital Leuven; Department of Human Genetics (A.S.), KU Leuven, Belgium; Department of Neurology (A.P.), University of Munich, Germany; Scientific and Technological Coordination Unit of the ANLIS Directorate (C.P.), National Administration of Laboratories and Institutes of Health, Argentina; Department of Neurodegenerative Diseases (T.G.), Center of Neurology and Hertie-Institute for Clinical Brain Research, University of Tübingen; German Center for Neurodegenerative Diseases (DZNE) (T.G.); Department of Neurology (K.C.), AZ Turnhout, Antwerp, Belgium; Neurology Unit and Stroke Center (F.B.), Hôpital Foch, Suresnes, France; Movement Disorder Division (K.M.), Johns Hopkins University, Baltimore, MD; and Psychological Medicine and Clinical Neurosciences (N.M.W.), MRC Centre for Neuropsychiatric Genetics and Genomics, Cardiff University School of Medicine, Cardiff University, UK.

## Abstract

**Objective:**

To delineate the natural history, diagnosis, and treatment response of Parkinson disease (PD) in individuals with 22q11.2 deletion syndrome (22q11.2DS), and to determine if these patients differ from those with idiopathic PD.

**Methods:**

In this international observational study, we characterized the clinical and neuroimaging features of 45 individuals with 22q11.2DS and PD (mean follow-up 7.5 ± 4.1 years).

**Results:**

22q11.2DS PD had a typical male excess (32 male, 71.1%), presentation and progression of hallmark motor symptoms, reduced striatal dopamine transporter binding with molecular imaging, and initial positive response to levodopa (93.3%). Mean age at motor symptom onset was relatively young (39.5 ± 8.5 years); 71.4% of cases had early-onset PD (<45 years). Despite having a similar age at onset, the diagnosis of PD was delayed in patients with a history of antipsychotic treatment compared with antipsychotic-naive patients (median 5 vs 1 year, *p* = 0.001). Preexisting psychotic disorders (24.5%) and mood or anxiety disorders (31.1%) were common, as were early dystonia (19.4%) and a history of seizures (33.3%).

**Conclusions:**

Major clinical characteristics and response to standard treatments appear comparable in 22q11.2DS-associated PD to those in idiopathic PD, although the average age at onset is earlier. Importantly, treatment of preexisting psychotic illness may delay diagnosis of PD in 22q11.DS patients. An index of suspicion and vigilance for complex comorbidity may assist in identifying patients to prioritize for genetic testing.

Parkinson disease (PD) is a complex neurodegenerative disorder. While many genetic factors have been identified that increase the risk to develop the disease,^1^ genetic testing is not part of general clinical practice.^2^ A recently discovered genetic risk factor, accounting for approximately 0.5% of patients with early-onset PD (EOPD), is the recurrent hemizygous 22q11.2 deletion associated with 22q11.2 deletion syndrome (22q11.2DS).^3,4^ Previously known as DiGeorge or velocardiofacial syndrome, 22q11.2DS is an underdiagnosed multisystem genetic condition that can include birth defects, intellectual and developmental disabilities, seizures, psychotic disorders, and endocrine abnormalities. The associated 22q11.2 deletion, detectable on clinical genetic testing, is estimated to be present in 1 in 3,000 live births.^5^

Although multiple case reports and 2 case series have been important in discovering the connection between PD and 22q11.2 deletions, and neuropathologic examination has shown classic loss of midbrain dopaminergic neurons with variable Lewy body pathology,^3^ it is essential to compile a larger sample with more comprehensive data to understand how this genetic subtype may be similar to—or different from—typical idiopathic PD and other genetic forms of PD. We therefore obtained clinical and neuroimaging data from physicians for all identified 22q11.2DS PD cases around the world, in order to delineate the natural history, diagnosis, and treatment response of this genetic subtype of PD. The results indicate that, while many features are similar to those of typical PD, an increased index of suspicion together with clinical clues from the patient's history may help prompt genetic testing for 22q11.2 deletion and prevent delayed diagnosis of PD.

## Methods

### Identification and characterization of cases

**Figure 1:**
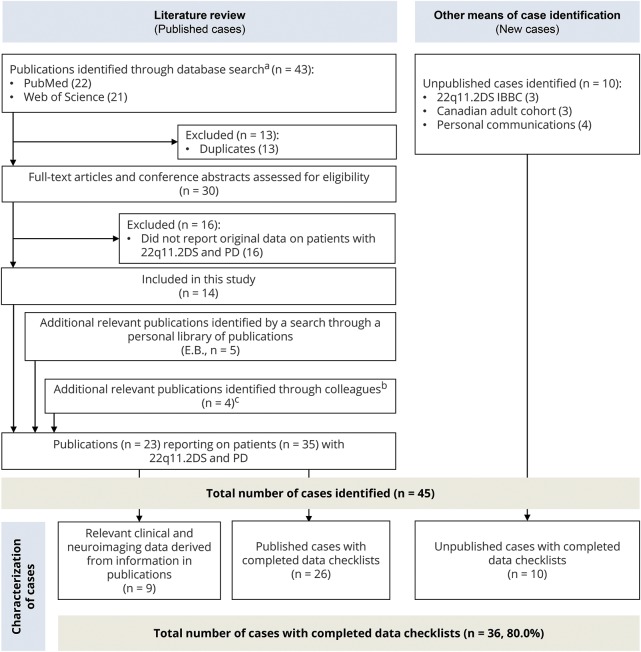
Study flow chart: Identification and characterization of patients with 22q11.2 deletion syndrome and Parkinson disease ^a^ Literature review performed on November 1, 2016. ^b^ One publication by our own group. ^c^ Two cases were reported during preparation of this article.^16,31^ 22q11.2DS IBBC = International Consortium on Brain and Behavior in 22q11.2 Deletion Syndrome.

We obtained clinical and neuroimaging data for 45 individuals with 22q11.2DS who met the inclusion criteria for this study: a molecularly confirmed 22q11.2 deletion involving the typically deleted region and PD defined as a clinical diagnosis by a neurologist,^2,5^ including bradykinesia and at least one of either rest tremor or rigidity. We excluded individuals deemed to have drug-induced parkinsonism or parkinsonism of unknown etiology.^6^ We used the standard EOPD definition of age at onset <45 years.^2^ We used comprehensive data forms to systematically collect anonymized clinical data on each patient with PD and 22q11.2DS (see appendix e-1, links.lww.com/WNL/A515); these were completed by the participating physicians using medical records or direct assessment for 26 (74.3%) of 35 previously reported cases identified through an extensive literature review and 10 unpublished cases identified through the International Consortium on Brain and Behavior in 22q11.2DS, a Canadian adult cohort, and personal communications (figure 1 [study flow chart]; table e-1, links.lww.com/WNL/A513 [published cases with PD and 22q11.2DS]; and appendix e-2 [results of literature search]).

### Standard protocol approvals, registrations, and patient consents

The requirement for informed consent for this retrospective study differed between participating countries; informed consent was obtained if required.

### Statistical analyses

Statistical analyses were conducted using IBM SPSS Statistics 22 for Windows (SPSS Inc., Chicago, IL). We used an independent-samples *t* test or Mann-Whitney *U* test to investigate differences in age at motor symptom onset, age at PD diagnosis, and time to clinically confirmed PD diagnosis, in male vs female patients, and in antipsychotic-naive patients vs those taking antipsychotic medication, as appropriate. We used the McNemar test to determine if there was an increase in prevalence of motor symptoms over the course of PD. We used a binary logistic regression analysis to investigate the association between sex and history of antipsychotic medication use and the presence of motor symptoms at presentation, and the association between sex and follow-up time and the prevalence of motor symptoms over the course of PD. All analyses were 2-tailed, with statistical significance defined as *p* < 0.05. We excluded cases missing values on a particular outcome for that analysis.

### Data availability

Anonymized data will be shared by request from any qualified investigator, only for purposes of replicating procedures and results.

## Results

### Diagnosis of 22q11.2DS, family history of PD, and other genetic factors

Patients tended to have a late diagnosis of the 22q11.2 deletion (mean age 40.2 ± 13.0 years, n = 38), with the genetic diagnosis in more cases occurring after (n = 24 [63.1%]) than before (n = 12 [31.6%]) PD motor symptom onset (n = 2 age at onset unknown). With respect to associated developmental features, there were just 11 (24.4%) patients with a congenital heart defect reported but 28 (62.2%) with intellectual disability, most in the mild range (table e-2, links.lww.com/WNL/A513).

As expected,^5^ most cases with information on inheritance status had a de novo 22q11.2 deletion (n = 16 of 19 [84.2%]). Three had maternally inherited deletions, including one rare mosaic deletion. Two female patients had an additional genetic finding of possible clinical relevance: 45,X[3]/46,XX[7] mosaic Turner syndrome, and a maternally inherited 3q29 duplication with unknown pathogenicity, respectively. Wilson disease was considered, but ruled out, in 2 male patients (at ages 30 and 46 years, respectively).

There were 4 cases (8.9%) reported to have a parent with PD: 1 where the parent had EOPD (31 years); 1 other also had 3 paternal second-degree relatives with a history of PD (1 with onset <50 years). A fifth patient had a brother with amyotrophic lateral sclerosis. The inheritance status of the 22q11.2 deletion was unknown in all of these 5 cases. Genetic testing was performed for known PD genes using various strategies for 17 patients, including 2 of the 5 with family history of neurodegenerative disease.^3,4^ The only finding was a missense mutation (HTRA2 p.G399S) of uncertain relevance to PD, inherited from an unaffected mother.^7^

### Sex distribution, age at motor symptom onset, and PD diagnosis

Of 45 individuals with 22q11.2DS-associated PD, 32 (71.1%) were male, indicating a typical PD sex distribution.^8^ Onset was early in 22q11.2DS-associated PD, as expected.^3,4,9^ For the 35 (81.4%) cases with data available, mean age at onset of motor symptoms was 39.5 ± 8.5 years, and 25 (71.4%) met EOPD criteria. Motor symptom onset on average was 2 years later in women, a nonsignificant sex difference (women [40.9 ± 8.2 years, n = 11], men [38.8 ± 8.7 years, n = 24]; *p* = 0.51). The mean age at PD diagnosis was 42.1 ± 9.0 years (n = 38 [88.4%]) with no significant sex difference (*p* = 0.30).

**Figure 2:**
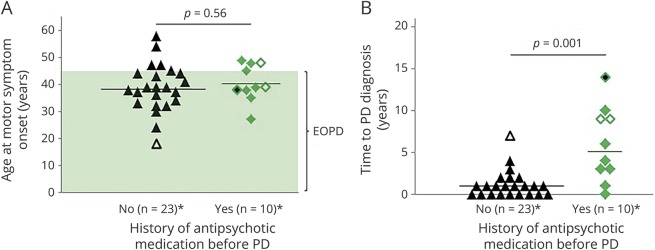
Antipsychotic medication and delay in diagnosis of Parkinson disease (PD) in 22q11.2 deletion syndrome *Complete data on age at motor symptom onset, age at PD diagnosis, and history of antipsychotic medication use were available for 33 cases (for n = 8 cases, antipsychotic status was uncertain). Two suspected PD cases receiving antipsychotic medication (see e-Methods, links.lww.com/WNL/A514) were excluded. (A) There was no difference in mean age at motor symptom onset (38.7 ± 8.9 vs 40.6 ± 6.9 years) between patients without and patients with a history of antipsychotic use. (B) However, the median time between motor symptom onset and a diagnosis of PD was shorter in antipsychotic-naive patients compared to those with a history of antipsychotic treatment. Triangle without fill = in the antipsychotic-naive group, the patient with the youngest age at motor symptom onset (18 years) had the longest time to diagnosis. Also, in this patient with reduced dopamine transporter (DAT) binding on imaging, bradykinesia could not be established formally due to cognitive impairment. Diamonds without fill = patients using clozapine before PD diagnosis. Diamond with black fill = in one patient, the neurologist deferred the PD diagnosis due to olanzapine use. Fourteen years after the onset of motor symptoms, DAT imaging showed the typical pattern of severely reduced striatal DAT binding. EOPD = early-onset (<45 years) PD.

Despite having similar ages at median motor symptom onset, individuals with a history of antipsychotic use had a longer median time to PD diagnosis (5, range 0–14 years) than antipsychotic-naive patients (1, range 0–7 years; *p* = 0.001, figure 2). Although clozapine is not expected to cause parkinsonism,^2^ there were 2 patients with a time to PD diagnosis of 9 years, on clozapine monotherapy for most of that time period.

### Motor symptoms, response to treatment, and mortality

Table 1 shows data available on motor symptoms in the onset year and at last assessment. For those with distribution of motor symptom onset documented (n = 30), the majority had asymmetric onset, including 20 of 22 (90.9%) antipsychotic-naive cases and 7 of 8 (87.5%) antipsychotic-exposed cases. A less typical finding at presentation was dystonia (table 1).^10^ Neither sex nor history of antipsychotic medication use appeared to affect the presence of motor symptoms.

**Table 1:**
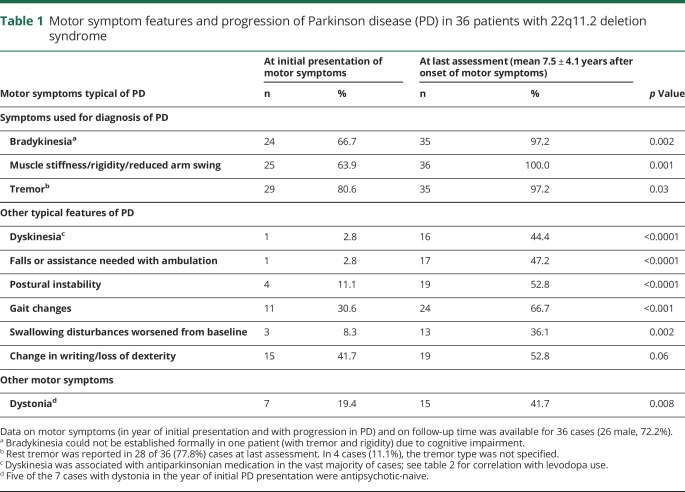
Motor symptom features and progression of Parkinson disease (PD) in 36 patients with 22q11.2 deletion syndrome

There was evidence of progression of motor symptoms over the course of PD with worsening for all typical features of PD except writing/loss of dexterity, where results were at the trend level (table 1). Neither presence of dystonia nor dyskinesia at last assessment was associated with sex or duration of follow-up (data not shown). Dyskinesia, however, was less likely to emerge with PD progression in women than men (odds ratio 0.10, 95% confidence interval 0.01–0.91; *p* = 0.04).

Most patients received typical PD treatments (table e-3, links.lww.com/WNL/A513) with response reported as positive (table 2). Polypharmacy and deep brain stimulation were fairly common (table 2).

**Table 2:**
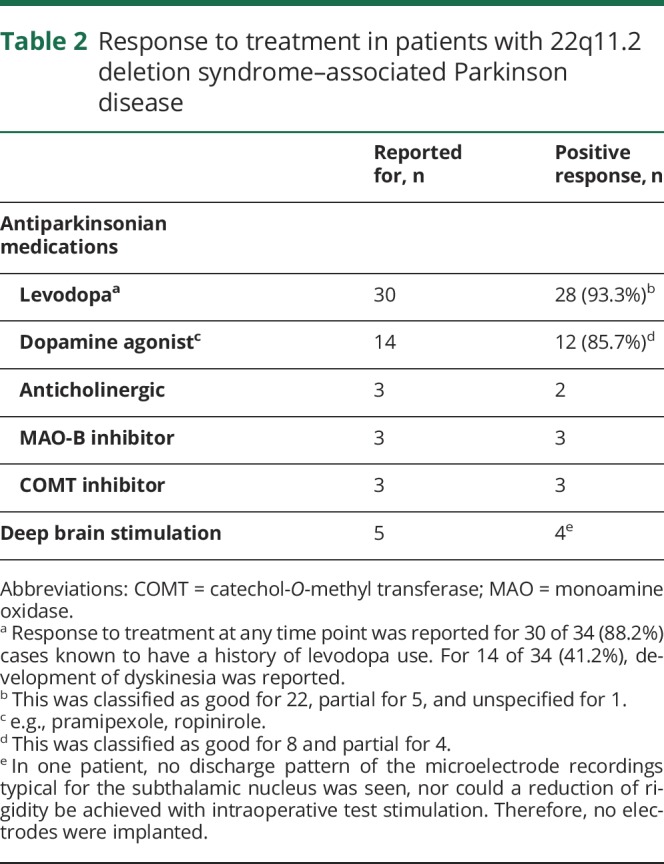
Response to treatment in patients with 22q11.2 deletion syndrome–associated Parkinson disease

Seven patients had died, 3 of whom met EOPD criteria. Median age at death was 56 years (range 42–61) after a median time from onset of motor symptoms of 9 years (5–18) for the 6 patients with data. There were only 3 patients with known cause of death: pneumonia (n = 2) and cardiac failure (n = 1).

### Neurologic and psychiatric symptoms

Before PD onset, there were 11 (24.4%) patients with a history of a psychotic disorder, and 14 (31.1%) with mood or anxiety disorder, in line with expectations for adults with 22q11.2DS.^11,12^ Fifteen (33.3%) patients had a lifetime history of seizures, a somewhat greater proportion than in a recent survey of 22q11.2DS (15.8%).^13^

With progression of PD, symptoms of psychosis, anxiety, or depression emerged in 7 (20.6%), 6 (17.6%), and 6 (18.2%) patients with no history of these, respectively. Emergence of cognitive decline was reported in 8 (17.8%) cases. Other emerging symptoms included impulse control disorders (n = 6), emotional lability (n = 3), altered sleep or eating habits (n = 3), confusion (n = 1), and self-injurious behavior (n = 1). Emergence of psychiatric symptoms with progression of PD was attributed to antiparkinsonian medication in 8 patients. There were only 10 (22.2%) cases with no known history of any lifetime neuropsychiatric disorder or symptoms at last assessment or death.

### Dopaminergic imaging

Twenty (44.4%) patients had presynaptic dopaminergic imaging results available. Of 18 patients with dopamine transporter (DAT) SPECT imaging results, 17 showed typical findings of reduced (contralateral or bilateral) striatal binding.^4,9,14,15^ This included 6 (33.3%) patients taking antipsychotic medication. One of the 18 patients showed a marked loss of striatal DATs in the ipsilateral putamen, and a slight reduction in binding in the contralateral caudate nucleus.

Two patients had data from a scientific study using PET and ^11^C-dihydrotetrabenazine ([^11^C]DTBZ), a radioligand that binds to the presynaptic vesicular monoamine transporter 2.^16^ One patient showed the typical pattern of severely reduced striatal [^11^C]DTBZ binding.^16^ The patient with the next lowest striatal binding levels among the patients with 22q11.2DS studied, in the lower range of that for the healthy control group, was a 55-year-old man with parkinsonism.^6,16–18^ He subsequently had further decline in motor and cognitive functioning, and at age 57 years, demonstrated an unequivocal improvement in motor symptoms following levodopa treatment, and was deemed to meet criteria for PD.

## Discussion

This international collaborative study is the largest to date on 22q11.2DS-associated PD, providing new data on the clinical presentation, progression, and treatment response. The results (summarized in table 3) suggest that a male excess and the main clinical features of PD, including response to levodopa with development of dyskinesia in a high proportion of patients, would be indistinguishable from idiopathic PD.^8,19^ The majority of the cases met EOPD criteria. We note however that given a median age at death in 22q11.2DS in the mid-40s,^20^ many patients may not live long enough to develop PD. Congenital or other later onset features could prompt clinicians to consider genetic testing for the 22q11.2 deletion, especially in individuals with early-onset PD, early dystonia, a history of seizures, and neurodevelopmental disorders such as schizophrenia or intellectual disability.

**Table 3:**
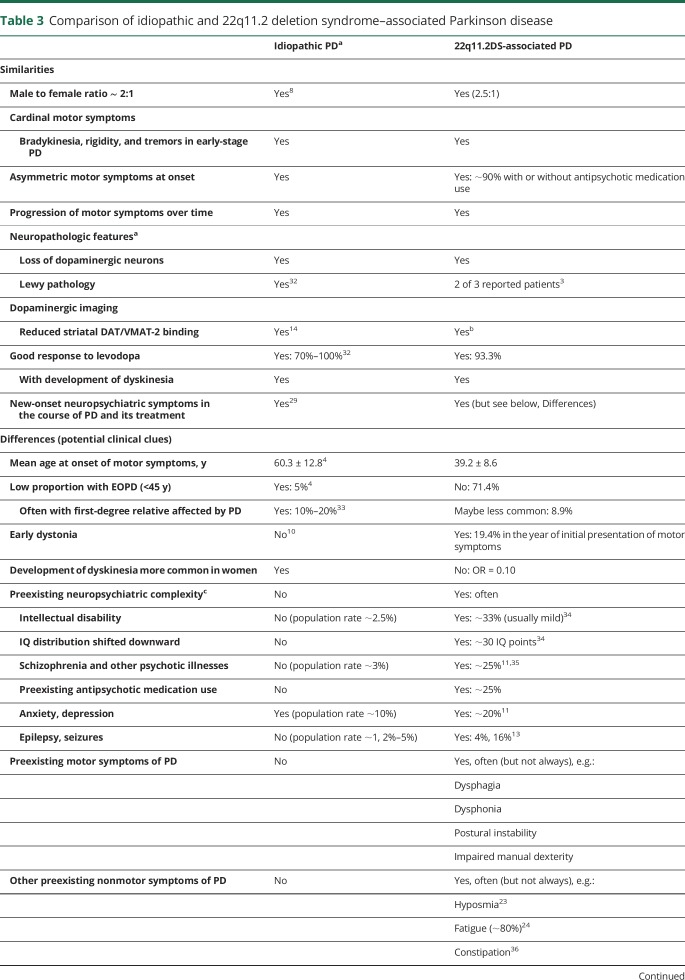

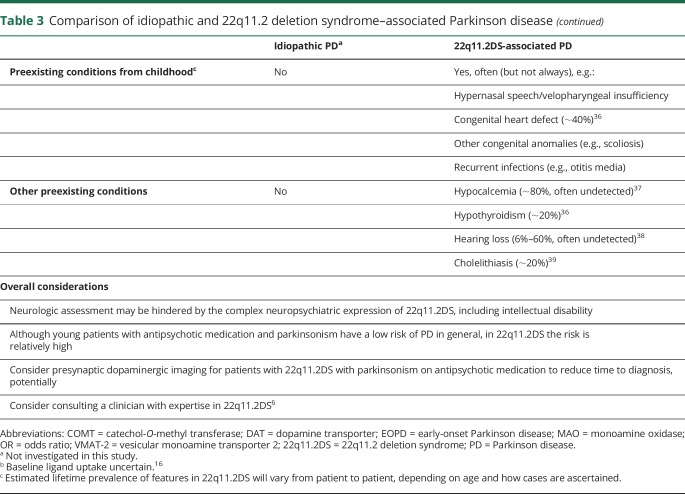
Comparison of idiopathic and 22q11.2 deletion syndrome–associated Parkinson disease

Patients exposed to antipsychotic treatment were diagnosed with PD later than antipsychotic-naive patients, despite the fact that few had symmetric parkinsonism.^21^ Some have proposed that nonmotor features including fatigue and hyposmia could help distinguish between medication-induced parkinsonism and PD.^22^ However, we did not assess these features in this study and believe them unlikely to be helpful as they are common manifestations of 22q11.2DS itself.^16,23,24^

The DAT imaging findings suggest that dopaminergic imaging, where available, may be helpful in the differentiation of PD from nondegenerative 22q11.2DS-related parkinsonism.^25^ This is particularly important when one considers that ∼25% of 22q11.2DS patients will need antipsychotic treatment.^26^ Potentially complicating the interpretation of imaging results for individuals with 22q11.2DS, however, are observations of paradoxical elevated striatal [^11^C]DTBZ and [^18^]F-PRO4.MZ (a DAT ligand) binding levels in some adults.^16^ This emphasizes the need for longitudinal imaging studies, and neuropathologic studies, to help delineate the dopaminergic mechanisms and trajectory of 22q11.2DS.^27^

Neuropsychiatric disorders are common features in 22q11.2DS.^11^ Nevertheless, a significant proportion of the patients in this study demonstrated emergence of psychotic symptoms with progression of PD. It remains unclear to what extent symptoms such as cognitive decline, psychosis, anxiety, and depression are due to PD,^28,29^ the 22q11.2 deletion,^26^ and/or effects of antiparkinsonian medications. Given the complex neuropsychiatric expression, including intellectual disability, other movement disorders,^6,16^ and the multisystem nature of 22q11.2DS,^5,26^ optimal management of 22q11.2DS-associated PD would involve collaboration between a movement disorders neurologist and specialist in 22q11.2DS.

If a 22q11.2 deletion is suspected, standard clinical microarrays will detect this structural change.^5^ It is important to realize that currently available PD genetic diagnostic panels do not include the 22q11.2 deletion. However, as for other PD-related mutations, much remains to be known about interacting factors that may contribute to the risk for PD imparted by a 22q11.2 deletion.^30^ Absence of an affected relative would not affect decision-making for genetic testing; the 22q11.2 deletion occurs as a spontaneous (de novo) mutation in most individuals.^30^

The strengths of the study include the collaborative nature of the work and the large number of patients, given that both EOPD and 22q11.2DS are relatively uncommon conditions. There were, however, several limitations. First, we acknowledge the lack of a typical PD comparison group. Second, publication bias has to be considered. For example, it is conceivable that patients with less typical PD or uncertain PD diagnosis, yet with true PD, are underrepresented. Third, it cannot be ruled out that handling of conflicting, ambiguous, missing, or unknown data may have influenced the study results. Fourth, survey responders may have interpreted definitions of variables differently. Fifth, there was a wide range of follow-up time from 0 to 21 years. Sixth, physicians did not systematically obtain clinical information on non-neurologic comorbidities; therefore we opted not to report on other 22q11.2DS-associated comorbidities (table e-2, links.lww.com/WNL/A513) in this study.

Further prospective clinical, neuropathologic, molecular, and animal studies promise to help clarify the pathogenesis of this molecular subtype of PD and indicate how well 22q11.2DS-PD could act as a genetic model for other forms of PD.

## Glossary

22q11.2DS: 22q11.2 deletion syndrome
DAT: dopamine transporter
EOPD: early-onset Parkinson disease
PD: Parkinson disease
